# Specification of haematopoietic stem cell fate via modulation of mitochondrial activity

**DOI:** 10.1038/ncomms13125

**Published:** 2016-10-12

**Authors:** Nicola Vannini, Mukul Girotra, Olaia Naveiras, Gennady Nikitin, Vasco Campos, Sonja Giger, Aline Roch, Johan Auwerx, Matthias P. Lutolf

**Affiliations:** 1 Laboratory of Stem Cell Bioengineering, Institute of Bioengineering, School of Life Sciences and School of Engineering, Ecole Polytechnique Fédérale de Lausanne (EPFL), CH-1015 Lausanne, Switzerland; 2 Laboratory of Regenerative Hematopoiesis (GR-NAVEIRAS), Institute of Bioengineering, School of Life Sciences, Ecole Polytechnique Fédérale de Lausanne (EPFL), CH-1015 Lausanne, Switzerland; 3 Departments of Medicine and Oncology, Centre Hospitalier Universitaire Vaudois (CHUV), CH-1011 Lausanne, Switzerland; 4 Laboratory of Integrative and Systems Physiology, Institute of Bioengineering, School of Life Sciences, Ecole Polytechnique Fédérale de Lausanne (EPFL), CH-1015 Lausanne, Switzerland; 5 Institute of Chemical Sciences and Engineering, School of Basic Science, Ecole Polytechnique Fédérale de Lausanne (EPFL), CH-1015 Lausanne, Switzerland

## Abstract

Haematopoietic stem cells (HSCs) differ from their committed progeny by relying primarily on anaerobic glycolysis rather than mitochondrial oxidative phosphorylation for energy production. However, whether this change in the metabolic program is the cause or the consequence of the unique function of HSCs remains unknown. Here we show that enforced modulation of energy metabolism impacts HSC self-renewal. Lowering the mitochondrial activity of HSCs by chemically uncoupling the electron transport chain drives self-renewal under culture conditions that normally induce rapid differentiation. We demonstrate that this metabolic specification of HSC fate occurs through the reversible decrease of mitochondrial mass by autophagy. Our data thus reveal a causal relationship between mitochondrial metabolism and fate choice of HSCs and also provide a valuable tool to expand HSCs outside of their native bone marrow niches.

## Abstract (web summary)

Haematopoietic stem cells rely on glycolysis for their energy demands but whether this affects their fate is unknown. Here, the authors show that forcing the cells to rely on glycolysis is important for self-renewal and that this involves a reduction in mitochondrial mass.

## Graphical abstract

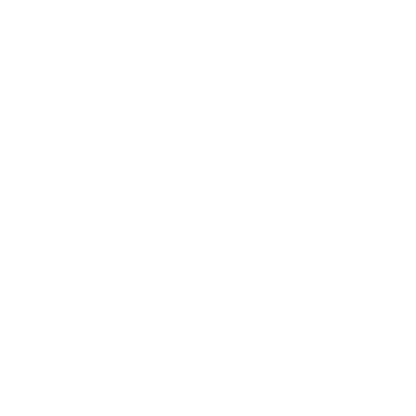

The maintenance of the blood system is ensured by a pool of HSCs residing in hypoxic niches in the bone marrow (BM)^1^. These unique cells are capable of lifelong self-renewal and commitment to multipotent progenitors (MPP). For many decades, HSCs have been successfully used for treating haematological and immune diseases. However, their limited number, especially when isolated from umbilical cord, prevents a more reliable and broader application of HSC-based therapies^2^,^3^,^4^. Despite recent notable success stories^5^,^6^, many attempts to propagate HSCs *in vitro* have failed, primarily because self-renewal and *in vivo* regenerative capacity is rapidly lost in culture.

Recent studies have shown that the change in cell identity and function during early HSC commitment involves a profound alteration in the metabolic program of the cells. Long-term HSCs (LT-HSCs) are mostly quiescent and tend to produce energy preferentially by anaerobic glycolysis^1^,^7^,^8^, which has been linked to their residence in low oxygen niches^9^,^10^. In contrast, the stem and progenitor cell types that produce blood and have a reduced self-renewal ability (that is, short-term HSCs and rapidly proliferating MPPs) generate ATP primarily in the mitochondria by oxidative phosphorylation (OXPHOS)^7^,^11^. The distinct metabolic program of LT-HSCs appears to play a critical role in maintaining their long-term *in vivo* function, presumably because the reduced mitochondrial respiration protects the cells from cellular damage inflicted by reactive oxygen species (ROS) in active mitochondria^12^,^13^,^14^,^15^,^16^.

The metabolic switch that occurs during the earliest step of adult haematopoiesis suggests a direct role of mitochondria in regulating HSC fate. This hypothesis is supported by work demonstrating that a metabolic transducer, the tumour suppressor and glucose sensor Lkb1 is crucial for HSC maintenance^16^,^17^,^18^,^19^. Moreover, autophagy, through which cells can modulate mitochondrial numbers, has been shown to improve HSC maintenance^20^. However, whether the metabolic state of HSCs is more than an adaptation to an extreme microenvironment in the BM, and perhaps linked to the ability to execute a particular cell fate choice, is currently not known.

Here we used the mitochondrial activity as a surrogate for the metabolic state of HSCs. Using *in vivo* multi-lineage blood reconstitution assays, we show that long-term self-renewal activity is restricted to phenotypic HSC subpopulations having lower mitochondrial activity. By comparing mitochondrial activity distributions of HSCs separated by their cell cycle phase, we find that during homeostasis as well as under acute stress, quiescent and cycling HSCs have relatively similar mitochondrial activity profiles. This shows that the distinct metabolic programs of HSCs are rather indicative of fate choice (that is, self-renewal versus commitment) and not *per se* a hallmark of the quiescent (versus activated) state. Indeed, *in vitro*, in heterogeneous HSC expansion cultures, divisional tracking experiments show that actively self-renewing HSCs retain a low mitochondrial activity, in marked contrast to differentiating cells that have activated mitochondria. Finally, under differentiation culture conditions, the chemical uncoupling of the electron transport chain forces HSC to self-renew, a process that is accompanied by a reversible decrease of mitochondrial mass by autophagy. Collectively, our experiments reveal an intriguing relationship between HSC metabolism and fate decision-making.

## Results

### HSCs show low mitochondrial activity and mass

To establish a reliable marker for tracking the metabolic state of HSCs undergoing different fate choices, we first employed flow cytometry and confocal microscopy to analyse the mitochondrial activity and mass of phenotypically defined HSC and MPP populations that were isolated by the most commonly used combinations of surface markers^21^,^22^ (Supplementary Fig. 1). We used tetramethylrhodamine methyl ester (TMRM), a cell-permeable dye that is readily sequestered by active mitochondria and reports mitochondrial polarization that correlates with the level of OXPHOS in a cell^23^,^24^. Each population displayed a distinct level of TMRM intensity, giving a stepwise increase from the most primitive to the most committed population as shown by flow cytometry (Supplementary Fig. 1a,b) and microscopy-based read-outs (Supplementary Fig. 1c,d). The mitochondrial activity was lowest in the LT-HSC population (Supplementary Fig. 1b), which was suspected based on recent studies reporting a preferential use of glycolysis in LT-HSCs^7^,^11^,^25^, and became barely detectable by confocal microscopy (Supplementary Fig. 1c,d).

We then asked whether HSCs and MPPs also show differences in their mitochondrial mass. To this end, we employed an R26-mito-EGFP mouse line^26^ ubiquitously expressing enhanced green fluorescent protein (EGFP) in the mitochondria of each cell. An analysis of single cells isolated from these mice by spinning disk microscopy revealed the lowest level of mitochondrial mass in the LT-HSC compartment (Supplementary Fig. 1e,f). This is in accordance with previous work showing that blocking HSC differentiation by TSC1-mediated mTOR pathway inhibition is accompanied by a decrease in mitochondrial mass^15^. Additionally, FOXO3, a transcription factor critical for the LT-HSC self-renewal^27^, promotes mitochondrial mass reduction^28^. Thus, these results confirm that under homeostatic conditions, freshly isolated LT-HSCs are marked by both low mitochondrial activity and mass.

### Low mitochondrial activity marks LT-HSCs

**Figure 1:**
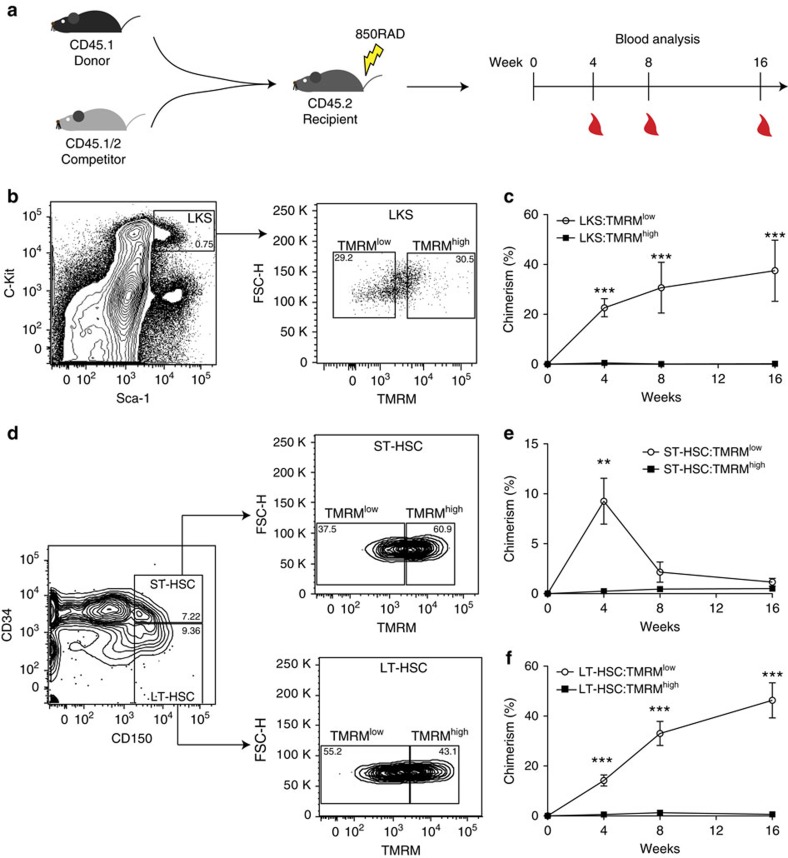
Multi-lineage reconstitution capacity is restricted to the low mitochondrial activity cell fractions. (**a**) Competitive transplantation strategy used to assess multi-lineage blood reconstitution levels from peripheral blood after 4, 8 and 16 weeks. (**b**,**c**) Within LKS, which contain all multipotent stem and progenitor cells in the BM, long-term multi-lineage HSC function is restricted to TMRM^low^ cells (LKS:TMRM^low^) (*n*=8 for each condition; error bar: s.e.m.; *t*-student, ***P<0.001). (**d**,**e**) In the phenotypically defined ST-HSC compartment, stemness is restricted to TMRM^low^ cells (ST-HSC:TMRM^low^) (*n*=9 for each condition; error bar: s.e.m.; *t*-student, **P<0.01). (**d**,**f**) In the phenotypically defined LT-HSC compartment, stemness is restricted to TMRM^low^ cells (LT-HSC:TMRM^low^) (*n*=9 for each condition; error bar: s.e.m.; *t*-student, ***P<0.001).

Using *in vivo* multi-lineage blood reconstitution assays, we next used phenotypically defined LKS (a population that contains all multipotent stem and progenitor cells in the BM, thus also the putative HSCs), ST- or LT-HSCs to test to which extent mitochondrial activity levels could report stem cell function (Fig. 1). First, we focused on LKS and utilized FACS to isolate two cell fractions within the LKS compartment characterized by low (LKS:TMRM^low^) and high (LKS:TMRM^high^) TMRM intensity levels. Then, we transplanted these two metabolically different cell populations into lethally irradiated mice by using a double congenic allelic system (Fig. 1a). Long-term multi-lineage blood reconstitution analysis showed that within the LKS population, only cells with low TMRM intensity (that is, LKS:TMRM^low^) permitted long-term multi-lineage reconstitution (Fig. 1b,c). Therefore, employing a metabolic read-out along with the existing surface marker repertoire allows purification of cells with long-term reconstitution capacity from a poorly defined population (LKS) consisting mainly of MPPs.

Then, using the same sorting strategy on ST-HSCs, setting the TMRM gates as shown in Supplementary Fig. 2, we compared the ability of ST-HSC:TMRM^low^ and ST-HSC:TMRM^high^ to reconstitute the blood system (Fig. 1d,e). Strikingly, within the ST-HSC population, the capacity to reconstitute the blood system was almost exclusively restricted to the TMRM^low^ fraction (Fig. 1e). Moreover, sorted LT-HSCs (Fig. 1d) could similarly be separated into two functionally very distinct populations. Only the population with low mitochondrial activity (LT-HSC:TMRM^low^, corresponding to ∼55% of the population) was capable of long-term multi-lineage reconstitution, whereas LT-HSC:TMRM^high^ cells completely failed to do so (Fig. 1f). Importantly, propidium iodide staining did not show any measurable difference in viability between the four populations (Supplementary Fig. 3), and BM isolated based on differential TMRM levels did not show significant differences in engraftment capability (Supplementary Fig. 4), ruling out that the lack of engraftment was a result of differential cell death. Moreover, we compared the homing capacity of transplanted cells and did not find any difference between HSCs isolated based on differential mitochondrial activity levels (Supplementary Fig. 5).

Our *in vivo* data thus reveal a striking functional heterogeneity in phenotypically defined HSCs. The observation that LT-HSCs with activated mitochondria (that is, LT-HSC:TMRM^high^) do not show a significant blood reconstitution suggests that these cells may not be hierarchically related to ‘true' LT-HSCs. They may instead represent HSCs that give rise to long-term lineage-restricted progenitor cells, as shown by *in vivo* single-cell multi-lineage reconstitution assays performed on the same immunophenotypes^29^. To test whether TMRM^low^ versus TMRM^high^ cells within the LT-HSC compartment contain different levels of such lineage-biased progenitor cells, we used CD41, a marker for megakaryocytes, to further separate the two phenotypes. Previous studies have shown that CD41+ cells in the HSC compartment have a strong myeloid bias^30^ and only short- or intermediate-term repopulation activity^29^. In our hands, LT-HSC:TMRM^low^ cells comprise ∼70% of cells that are negative for CD41, in marked contrast to LT-HSC:TMRM^high^ cells, of which ∼50% are CD41 positive (Supplementary Fig. 6). Therefore, the observed functional heterogeneity may at least be partially explained by the presence of CD41+ megakaryocyte progenitors that might have different mitochondrial activity levels compared with functional LT-HSCs.

### Low mitochondrial activity marks rare self-renewing HSCs

After establishing low mitochondrial activity as a functional discriminator of stemness, we tested whether this read-out could be used to distinguish self-renewing from differentiating HSCs in heterogeneous bulk cultures. To test whether TMRM-based read-outs could be used to separate self-renewal from differentiation divisions, 200 LT-HSCs from CD45.1 donor mice were isolated based on surface marker expression and expanded *in vitro* in serum-free medium containing a cocktail of the self-renewing factors angiopoietin-like protein, insulin-like growth factor binding protein 2, stem cell factor, fibroblast growth factor 1 and thrombopoietin^31^ (Supplementary Fig. 7). After 5 days in culture, the cells were resorted by FACS based on TMRM^low^ and TMRM^high^ phenotypes, and transplanted into lethally irradiated CD45.2 recipient mice (Supplementary Fig. 7a). Of note, we found that the TMRM intensities of cultured cells are substantially different from those of freshly isolated cells (Fig. 1b), likely reflecting an influence of cell culture. Nonetheless, consistent with our previous results using low TMRM fluorescence to directly isolate functional HSCs *in vivo*, a low TMRM signal was also predictive of the long-term blood reconstitution capacity of cultured HSCs (Supplementary Fig. 7b). Analysis of peripheral blood chimerism 4 months after grafting the cultured HSCs showed that TMRM^low^ cells induce significantly higher long-term multi-lineage blood reconstitution levels compared with cells that had a high TMRM signal. This suggested that self-renewing HSCs in culture can be detected based on the same metabolic hallmark as freshly isolated HSCs from the BM.

**Figure 2:**
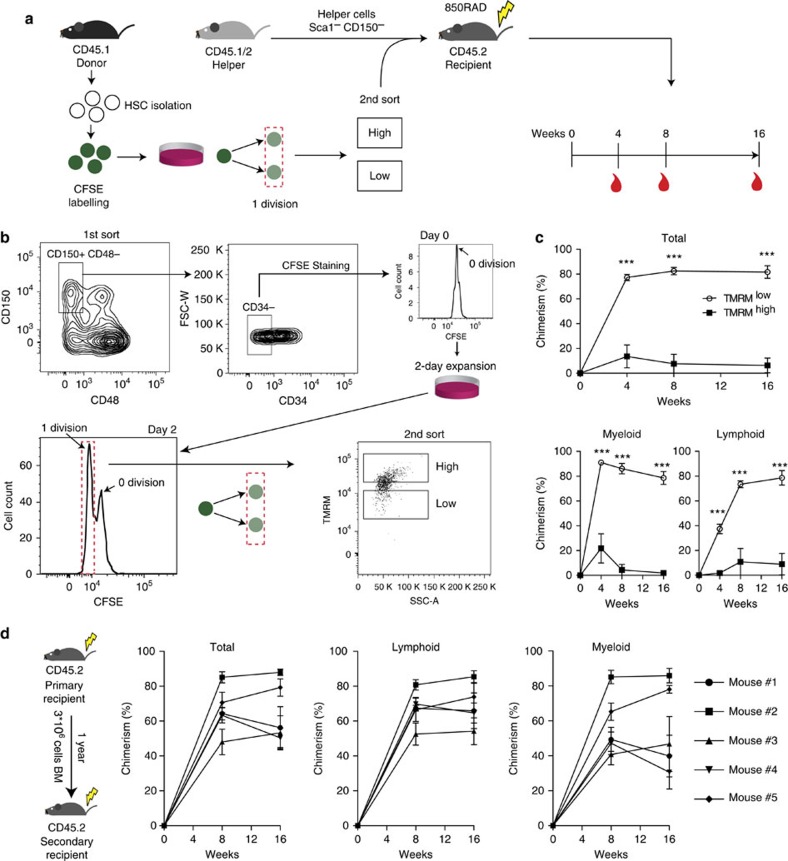
A low mitochondrial activity marks self-renewing HSCs. (**a**) Experimental paradigm to assess mitochondrial activity as a discriminator of self-renewing from differentiating LT-HSCs in culture. Freshly isolated LT-HSCs were labelled with CFSE. At the end of the culture, cells that divided one time were further sorted into TMRM^low^ and TMRM^high^ phenotypes and transplanted into lethally irradiated recipient mice together with helper cells. Blood reconstitution was assessed at 4, 8 and 16 weeks. (**b**) CFSE-labelled LT-HSCs were cultured under expansion conditions for 2 days and progeny that underwent one division were sorted into TMRM^low^ and TMRM^high^ phenotypes, and 100 cells of each population (along with 2 million helper cells) were transplanted into lethally irradiated recipients. (**c**) The TMRM^low^ fraction of the first generation of daughter cells (that is, dividing one time) exhibited strikingly higher long-term multi-lineage blood reconstitution efficiency compared with TMRM^high^ cells, providing evidence for self-renewing versus differentiating HSC divisions in culture (*n*=10 for each condition). Assessment of blood chimerism is shown for total blood (top panel) as well as the lymphoid and myeloid lineages (bottom panels; error bar: s.e.m.; *t*-student, ***P<0.001). (**d**) BM derived from each of the TMRM^low^ primary recipients (from **c**) was injected into four secondary recipient mice after 1 year of the primary transplant. Blood chimerism (average of four secondary recipients corresponding to each primary recipient) show long-term multi-lineage reconstitution in secondary transplants (error bar: s.e.m.).

**Figure 3:**
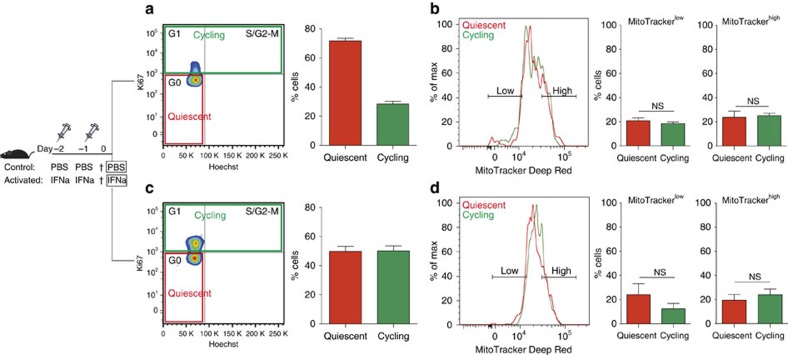
Quiescent and cycling HSC populations in the native niche have comparable mitochondrial activity levels. (**a**) Cell cycle analysis using Ki67 and Hoechst staining on freshly isolated HSCs (LKS CD150+ CD48− CD34−) indicate that more than 70% of the cells are in a quiescent state (G_0_, red), with the remaining cells cycling (G_1_+S/G_2_-M, green) (*n*=3). (**b**) Flow cytometry analysis of quiescent and cycling HSCs based on mitochondrial activity labelled with MitoTracker Deep Red. Both, quiescent and cycling HSCs show overlapping mitochondrial activity profiles. The proportion of low and high mitochondrial activity cells within the quiescent and cycling HSC populations is similar (MitoTracker^low^: *P*=0.44, MitoTracker^high^: *P*=0.81; *n*=3; error bar: s.e.m., *t*-student). (**c**) IFN-α stimulation results in *in vivo* activation of HSCs as demonstrated by Hoechst/Ki67 staining (*n*=3). (**d**) Flow cytometry analysis shows overlapping MitoTracker profiles of quiescent and cycling HSCs in IFN-α condition. Similarly, the proportion of MitoTracker^low^ and MitoTracker^high^ cells in quiescent and cycling HSCs remains comparable (MitoTracker^low^: *P*=0.32, MitoTracker^high^: *P*=0.54; *n*=3; error bar: s.e.m., *t*-student), suggesting that mitochondrial activity is independent of HSC cell cycle state even under acute stress conditions.

However, since it is known that a small fraction of cultured HSCs can maintain their stem cell potential by remaining quiescent even after prolonged time in culture (for example, refs 32, 33), the reconstitution from TMRM^low^ cells seen in Supplementary Fig. 7 might have come from non-dividing HSCs. To exclude this possibility and unequivocally identify self-renewing cells in these bulk cultures, we created a cell-labelling strategy that allowed us to track the precise number of divisions a ‘mother' HSC had undergone in culture (Fig. 2a,b). Freshly isolated LT-HSCs were uniformly labelled with carboxy-fluoresceinsuccinimidyl ester (CFSE), a live cell-permeable dye that allows cell division tracking^34^. With this tool, we FACS-sorted cultured HSC daughter cells that underwent precisely one division after 2 days and then further separated them into TMRM^low^ and TMRM^high^ subpopulations (Fig. 2b, lower panels). Hundred cells of each population were transplanted into lethally irradiated mice and long-term multi-lineage reconstitution was analysed up to 16 weeks later. In line with our previous findings, long-term multi-lineage reconstitution was once again restricted to the TMRM^low^ subpopulation (Fig. 2c). Furthermore, secondary transplantation showed long-term engraftment of the TMRM^low^ fraction (Fig. 2d). These data establish low mitochondrial activity as a read-out for self-renewal divisions of HSCs *in vitro*. Moreover, our experiments reveal that low mitochondrial activity is a hallmark not only of quiescent LT-HSC *in vivo*, as could be hypothesized based on their metabolic state of preferentially undergoing anaerobic glycolysis^9^,^10^, but also of cycling stem cells that have undergone a *self-renewing* cell division *in vitro*. Notably, a cell cycle phase analysis of HSCs *in vivo* showed that quiescent and cycling LT-HSCs have similar levels of mitochondrial activity, both under homeostatic conditions (Fig. 3a,b) and when stimulated by interferon-alpha (IFN-α) to exit dormancy^35^ (Fig. 3c,d) and maintaining stem cell potential (Supplementary Fig. 8). In the latter experiments, analysis of mitochondrial activity combined with cell cycle phase analysis was performed by MitoTracker Deep Red staining that persists in the cells after fixation^10^,^36^ and gave consistent read-outs on the metabolic profiles of the different haematopoietic compartments (Supplementary Fig. 9).

### HSC fate specification by electron transport chain uncoupling

**Figure 4:**
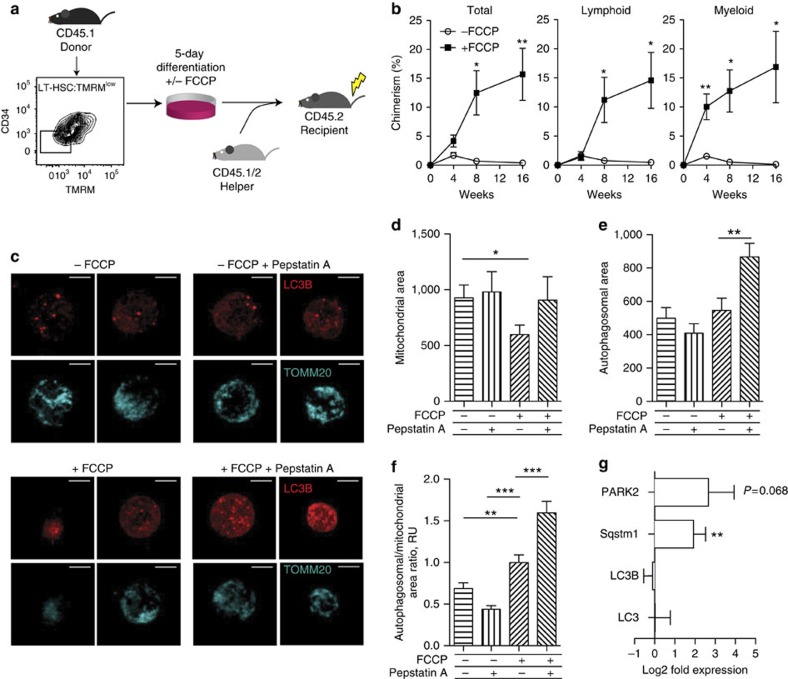
Modulation of mitochondrial metabolism alters HSC fate through autophagy. (**a**) LT-HSC:TMRM^low^ (LKS CD150+ CD34− TMRM^low^) cultured for 5 days under differentiation conditions in the presence or absence of FCCP were transplanted in lethally irradiated recipient mice together with 2*10^6^ helper cells. (**b**) Cells cultured in the presence of FCCP show high levels of multi-lineage reconstitution, in contrast to controls lacking FCCP that result in rapid differentiation (error bar: s.e.m.; *t*-student, **P<0.01 and *P<0.05). (**c**) Mitochondrial mass (TOMM20, cyan) and autophagosomes (LC3B, red) in different haematopoietic stem/progenitor cell populations. Images represent maximum intensity projections of the corresponding Z-stacks (0.28 μm step) Scale bar: 5 μm. (**d**–**f**) Quantification of the mito-EGFP (**d**), LC3B (**e**) and mito-EGFP/LC3B ratio intensity-thresholded areas (**f**). (**g**) Gene expression analysis on key autophagosomal and mitophagic genes was performed on LT-HSCs treated over 5 days with FCCP (error bar: s.e.m.; *t*-student, ***P<0.001, **P<0.01 and *P<0.05).

With a read-out identified to discriminate HSC self-renewal from differentiation, we then tested whether HSC fate could be manipulated by modulation of mitochondrial activity *in vitro* (Fig. 4). Specifically, we chose culture conditions that would rapidly push HSCs to differentiate into highly proliferative MPPs and asked whether blocking the establishment of a mitochondrial membrane potential would result in the maintenance of stemness (Fig. 4a). For that purpose, we used carbonyl cyanide-*p*-trifluoromethoxyphenylhydrazone (FCCP), which permeabilizes the inner mitochondrial membrane and disrupts its potential (Supplementary Fig. 10), to uncouple electron transport from ATP generation.

Long-term blood reconstitution assays show that LT-HSC:TMRM^low^ cultured for 5 days under differentiation-inducing conditions (SCF, Flt3, IL-3 and IL-6) and in the presence of FCCP exhibited substantially higher levels of long-term multi-lineage blood reconstitution in lethally irradiated recipient mice, compared with cells cultured in the absence of FCCP (Fig. 4b). By labelling HSCs with CFSE and tracking their divisional history, we could rule out that this effect was due to an induction of quiescence upon FCCP administration, since all cells in culture had divided multiple times (Supplementary Fig. 11). Therefore, by disrupting the inner mitochondrial membrane potential, HSCs that would normally rapidly differentiate can be converted to a state of self-renewing division. These data demonstrate that HSC self-renewal divisions can be executed independently of the establishment of a mitochondrial potential. Interestingly, rapidly dividing, self-renewing embryonic stem cells have mitochondria with a low mitochondrial membrane potential indicative of OXPHOS-independent metabolism^37^. Furthermore, during reprogramming of fibroblasts to the induced pluripotent stem cell state, a metabolic switch from OXPHOS to glycolysis is required^24^. Of note, LT-HSC:TMRM^high^ treated with FCCP failed to revert to functional stem cells (Supplementary Fig. 12), enforcing the hypothesis that LT-HSC:TMRM^high^ cells may be composed of more committed haematopoietic cells. Indeed, LT-HSC:TMRM^low^ are exclusively composed by CD48− cells, while the TMRM^high^ fraction contains both CD48− and CD48+ cells (Supplementary Fig. 13), as well as CD41+ cells (Supplementary Fig. 6).

### Chemical uncoupling increases autophagy in HSCs

FCCP is known to promote mitophagy^38^ and, in turn, autophagy has been demonstrated to be critical for LT-HSC self-renewal^27^,^28^,^39^. Accordingly, the inhibition of autophagy gives rise to an increase in the mitochondrial mass in LT-HSCs through an accumulation of mitochondria damaged by ROS^20^. Therefore, we postulated that the observed metabolic specification of HSC fate (Fig. 4b) could occur through a reversible decrease of mitochondrial mass via autophagy. To test this, we used spinning disk confocal imaging at single cell level to analyse whether the treatment of HSCs with FCCP under differentiation-inducing conditions is accompanied by a decrease of mitochondrial mass (TOMM20) and/or elevation of autophagy (LC3B) (Fig. 4c). The rate of autophagosomal turnover was assessed by blocking their lysosomal degradation by Pepstatin A for 4 h prior to fixation. This analysis showed that chemical uncoupling by FCCP drastically decreased the mitochondrial area as measured by TOMM20 in LT-HSC (Fig. 4d). This phenomenon is reversed in the presence of protease inhibitor Pepstatin A, which causes the accumulation of autophagosomes before mitochondrial autophagy can occur. Thus, while autophagosomal area as measured by LC3B immunofluorescence seems similar in FCCP-treated versus control HSCs (Fig. 4e), further treatment with protease inhibitor Pepstatin A shows a drastic increase in LC3B autophagosome accumulation in FCCP-treated cells, but not in control cells. This effect is especially clear, when the autophagosomal area is normalized to the mitochondrial area. The ratio between the autophagosomal and mitochondrial area is higher in FCCP-treated cells compared to control cells, and this effect is further emphasized by the inhibition of lysosomal degradation of the autophagosomes by Pepstatin A (Fig. 4f). Taken together, these results point to the reduction in mitochondrial mass in LT-HSCs upon FCCP treatment as a result of both increased autophagosome formation and accelerated lysosomal processing of the autophagosomes. Consistently, gene expression analysis revealed an increase in expression levels of the E3 ubiquitin ligase PARKIN (encoded by PARK2), involved in the ubiquitination of depolarized mitochondria^40^, and of the autophagic receptor Sqstm1^41^ (Fig. 4g). Therefore, the chemical uncoupling of the electron transport chain increases the level of autophagy in LT-HSCs, and the mitochondria serve as a primary target for the removal by autophagosomes.

## Discussion

Collectively, our data show that in HSCs, mitochondrial metabolism and function are intricately linked. Specifically, we demonstrate that the distinct metabolic program of HSCs is more than an adaptation to a specific microenvironment in the BM niche^9^,^10^, but seems sufficient to drive the decision of stem cells to undergo self-renewal or commitment. It should be noted, however, that the mitochondrial activity is a transient cell state that may strongly depend on endogenous conditions such as the microenvironment. Cells may not be readily interconvertible between high and low mitochondrial activity levels (Supplementary Fig. 12).

Using a gene-knockout approach, previous work has shown that loss of function of PTPMT1 phosphatase within the inner mitochondrial membrane is sufficient to block differentiation and promote self-renewal *in vivo*, apparently through the accumulation of phosphatidylinositol phosphate substrates that enhance endogenous UCP2 activity and lower mitochondrial aerobic metabolism^11^. However, loss of PTPMT1 failed to drive preferential HSC self-renewing divisions *in vitro*, presumably because of the oxidative stress imposed by classic culture conditions. Loss of PTPMT1 is also an irreversible treatment that results in the inability of stem cells to differentiate *in vivo*, causing abnormal expansion of the HSC compartment and ultimately a failure of haematopoiesis. Here we show that chemical uncoupling of the electron transport chain is sufficient to reversibly metabolically modulate HSCs via induction of autophagy, maintaining their capacity for proper differentiation and functional blood reconstitution.

We think that our approach could be useful to expand HSCs for clinical applications. Moreover, the availability of a functional marker for HSC self-renewal in the form of low mitochondrial potential as measured by TMRM, extensively validated here for both *in vitro* and *in vivo* applications, should facilitate investigations of molecular mechanisms of HSC fate decision-making.

## Methods

### Mice

C57Bl/6J and C57Bl/6J Ly5.1 were purchased from Charles River Laboratories International and maintained at the Center for Studying Living System (CAV) at EPFL in micro-isolator cages. Mice were provided continuously with sterile food, water and bedding. All *in vivo* procedures were carried out in accordance with the Swiss law and EPFL policies.

### Antibodies

The following antibodies were used: rat mAbs against 1/200 cKit (2B8) (Biolegend Cat. no. 105814), 1/100 Sca1 (D7) (Biolegend Cat. no. 108112), 1/100 CD150 (TC-15-12F12.2), 1/25 CD34 (RAM34), 1/100 CD48 (HM48-1) (Biolegend Cat. no. 103418), 1/200 CD45.2 (104) (Biolegend Cat. no. 109820), 1/200 CD45.1 (A20) (Biolegend Cat. no. 110706), 1/1,000 Gr1 (RB6-8C5) (Biolegend Cat. no. 108412), 1/750 F4/80 (BM8), 1/500 CD19 (6D5) (Biolegend Cat. no. 115507), 1/200 CD3 (17A2) (Biolegend Cat. no. 100206), 1/500 CD16/CD32 (2.4G2) (BD Cat. no. 553141). The antibodies were purchased from Biolegend, eBiosciences and BD (Becton, Dickinson and Company). A mixture of biotinylated mAbs against CD3, CD11b, CD45R/B220, Ly-6G, Ly-6C and TER-119 was used as lineage cocktail (BD).

### Flow cytometry and fluorescence activated cell sorting

Flow cytometry analysis of haematopoietic stem and progenitor cells was performed on freshly isolated BM. BM was extracted from crushed femora and tibia. Cell suspensions were filtered through a 70 μm cell strainer and erythroid cells were eliminated by incubation with red blood cells lysis buffer (eBioscences). Lineage-positive cells were removed with a magnetic lineage depletion kit (Miltenyi Biotech). Cell suspensions were stained with a panel of specific antibodies for stem and progenitor cells and analysed or FACS-sorted on a BD LSRII and BD FACS Aria II, respectively.

### Analysis of mitochondrial activity

Freshly isolated BM cells were incubated at 37 °C for 1 h with 200 nM TMRM (Invitrogen) and then stained with specific antibodies for the different haematopoietic stem/progenitor cell compartments. Labelled cells were FACS-sorted or analysed by flow cytometry. For confocal imaging, LT-HSC, ST-HSC, MPPs and committed progenitors were sorted and placed on adherent poly-L-lysine (PLL)-coated glass slides for 6 h. 20 nM TMRM was then added in the media and live cell images were acquired on a Leica SP5 confocal microscope. For MitoTracker Deep Red (Invitrogen) staining, cells were incubated at 200 nM for 1 h at 37 °C.

### Cell cycle staining

Sorted HSCs were fixed and permeabilized using Cytofix/Cytoperm plus kit (BD), according to the manufacturer's instruction. Fixed cells were then stained overnight with FITC Ki67 (BD) at 4 °C, and 10 min by Hoechst 33342 (Invitrogen).

### CFSE staining

Freshly sorted LT-HSCs were incubated for 20 min at 37 °C with 1:400 CFSE stock solution (Cayman chemicals; CFSE cell division assay kit). The cells were pelleted and resuspended in 1 ml of Stemline II (Sigma) containing 10% FBS for 20 min at 37 °C. Afterwards, the cells were washed twice with 1 ml of Stemline II (Sigma) and put in culture.

### HSC culture

HSCs were cultured under ‘expansion' conditions in U-bottom 96-well plates for 5 days. Cultures were maintained in Stemline II (Sigma) supplemented with 10 μg ml^−1^ Heparin (Sigma), 100 ng ml^−1^ SCF (R&D Systems), 2 ng ml^−1^ Flt3 ligand (R&D), 20 ng ml^−1^ TPO (R&D Systems), 10 ng ml^−1^ FGF-1 (Invitrogen), 500 ng ml^−1^ IGFBP2 (R&D Systems) and 100 ng ml^−1^ AngL-3 (R&D Systems)^31^. At the end of the culture period, cells were stained with TMRM and analysed or sorted by flow cytometry. To induce differentiation, HSCs were cultured in a basal medium (Stemline II containing 100 ng ml^−1^ SCF and 2 ng ml^−1^ Flt3 ligand) supplemented with 20 ng ml^−1^ IL-3 (R&D Systems) and 100 ng ml^−1^ IL-6 (R&D Systems). For some experiments, 5 μM carbonyl cyanide 4-(trifluoromethoxy)phenylhydrazone (FCCP) (Sigma) was added at the medium. FCCP stock solution was prepared by dissolving the powder in ethanol at 10 mM concentration.

### Transplantations

C57Bl/6 Ly5.2 female mice (8–12-weeks-old) were lethally irradiated on an X-ray irradiator at 850 rads in two split doses of 425 rads separated by 3–5 h and transplanted with donor cells derived from C57Bl/6 Ly5.1 mice and competitor cells derived from F1 C57Bl/6 Ly5.1/5.2 mice. For LKS transplants, 1,000 LKS (TMRM^low^ or TMRM^high^) donor cells were transplanted together with 250 × 10^3^ total BM competitor cells in recipient mice. For LKS CD150+CD34− (‘LT-HSC') and LKS CD150+ CD34+ (‘ST-HSC') transplants, 80 LT-HSC (TMRM^low^ or TMRM^high^) or 80 ST-HSC (TMRM^low^ or TMRM^high^) were transplanted together with 250 × 10^3^ total BM competitor cells in recipient mice. Peripheral blood was collected at 4, 8 and 16 weeks to determine the the percentage of chimerism. For transplantation of *in vitro*-cultured LT-HSCs, the progeny of 200 LT-HSCs cultured for 5 days were FACS-sorted based on their TMRM signal (TMRM^low^ or TMRM^high^) and transplanted together with 2 × 10^6^ helper cells. Helper cells were derived from BM of C57Bl/6 Ly5.1/5.2 mice that were depleted for Sca1 and CD150 positive cells (Miltenyi Biotech). Peripheral blood was collected at 4, 8 and 16 weeks to determine the percentage of chimerism. For the CFSE-TMRM transplants, LT-HSCs were sorted and stained for CFSE. At the end of a 2-day culture period, cell progeny having undergone one division were stained with TMRM and resorted based on TMRM^low^ and TMRM^high^ signals. Each recipient mouse was injected with 100 cells of either population together with 2 × 10^6^ helper cells. Peripheral blood was collected at 4, 8 and 16 weeks to determine the percentage of chimerism.

For transplants of cultured HSC exposed to the uncoupler FCCP, 100 LT-HSC:TMRM^low^ cells were cultured for 5 days under differentiation-inducing conditions in the presence or absence of 5 μM FCCP. FCCP was replenished every 24 h. Cell progeny were transplanted in lethally irradiated recipient mice together with 2 × 10^6^ helper cells. Peripheral blood was collected at 4, 8, 12 and 16 weeks to determine the level of chimerism.

For secondary BM transplantation, secondary C57Bl/6 Ly5.2 recipient mice were lethally irradiated as described above and transplanted with 3 million BM cells from the primary recipients. Peripheral blood was collected at 4, 8 and 16 weeks to determine the level of chimerism.

### HSC homing

C57Bl/6 Ly5.2 mice were lethally irradiated on an X-ray irradiator at 850 rads in two split doses of 425 rads separated by 3–5 h and transplanted with donor cells derived from C57Bl/6 Ly5.1/5.2. Four thousand LT-HSC (TMRM^low^ or TMRM^high^) were transplanted in recipient mice. BM was collected after 16 h to determine the percentage of homed donor-derived cells^42^.

### Analysis of mitochondrial mass and autophagy level

For the live cell mitochondrial mass measurements, freshly isolated BM cells from R26-Mito-EGFP mice were stained with specific antibodies for the different haematopoietic stem/progenitor cell compartments. Labelled cells were FACS-sorted and live cells analysed by spinning disk confocal microscopy on a Visitron CSU W1 microscope. Mitochondrial mass was quantified as the intensity-thresholded area of mito-EGFP signal. For the autophagy-level study, freshly isolated BM cells were stained with specific antibodies for the different haematopoietic stem/progenitor cell compartments. LT-HSC:TMRM^low^ cells were FACS-sorted, separated in halves and cultured in differentiation-inducing medium, defined as basal medium (Stemline II containing 100 ng ml^−l^ SCF and 2 ng ml^−1^ Flt3 ligand) supplemented with 20 ng ml^−1^ IL-3 (R&D Systems) and 100 ng ml^−1^ IL-6 (R&D Systems). Cells were cultured for 5 days either with addition of 5 μM FCCP or an equivalent amount of vehicle (EtOH). On the last day of culture, each population was separated in halves and further cultured for 4 h in the following conditions: FCCP-treated cells were either cultured with 5 μM FCCP and 1 μM Pepstatin A or with 5 μM FCCP and an equivalent amount of vehicle (DMSO), while the control vehicle-treated cells were either with vehicle (EtOH) and 1 μM Pepstatin A or with vehicle alone (EtOH+DMSO). After 4 h, the cells were fixed with 4% formaldehyde for 5 min at room temperature, permeabilized with MeOH for 5 min, washed with PBS pH 7.4 and blocked for 1 h with 2% goat serum in PBS pH 7.4 with slight agitation. The cells were incubated for an additional 2 h with the rabbit anti-LC3B (Abcam, ab51520) and mouse anti-TOMM20 (Abcam ab56783) primary antibodies with slight agitation, washed three times with PBS pH 7.4, incubated with goat anti-mouse Alexa488 and goat anti-rabbit Alexa647 secondary antibodies with slight agitation for 1 h and washed three times with PBS pH 7.4. Stained cells were imaged by spinning disk confocal microscopy on a Visitron CSU W1 microscope. Mitochondrial and autophagosomal mass were quantified as the intensity-thresholded area of TOMM20 and LC3B signal, respectively.

### *In vivo* activation of HSC

HSC were activated to exit dormancy by IFN-α treatment following published protocols^35^. Briefly, subcutaneous injections in C57Bl/6J mice were carried out with 10,000 U of IFN-α (R&D systems) 48 and 24 h prior to BM extraction. Control mice were injected with an equivalent volume of the vehicle (PBS+0.1% BSA).

### QPCR analysis

LT-HSCs were lysed after 5 days culture in lysis buffer from ZR RNA MicroPrep (Zymo Research) and RNA extraction was performed accordingly to the manufacturer's instruction. RNA was eluted and resuspended in 6 μl of H_2_O. Four microlitres of RNA was retro-transcribed to cDNA with Vilo SScript system (Invitrogen). Subsequently cDNA was diluted five times in water. For QPCR 1.5 μl of cDNA, 5 μl of Power Syber Green mastermix (Applied Biosystem) and 200 nM of primers were added to a final volume of 10 μl for each reaction. The reactions were performed on 7900HT system (Applied Biosystem). Primer sequences (5′-3′) are the following: LC3-F GTCACCCAGGCGAGTTACC; LC3-R TTACAGCGGTCGGCGAAG; Sqstm1-F GCTGAAGGAAGCTGCCCTAT; Sqstm1-R TTGGTCTGTAGGAGCCTGGT; Park2-F CCGAATCACCTGACGGTTCA; Park2-R TCTGGCTGCTTCTGAATCCC; Arbp-F AGATTCGGGATATGCTGTTGG; Arbp-R AAAGCCTGGAAGAAGGAGGTC.

### Statistics

Data were statistically analysed by Student's *t*-test, one-way ANOVA followed by Bonferroni's multiple comparison test and Mann–Whitney test.

### Data availability

The authors declare that all relevant data are contained within the article and Supplementary files, or are available from the authors on request.

## Additional information

**How to cite this article:** Vannini, N. *et al*. Specification of haematopoietic stem cell fate via modulation of mitochondrial activity. *Nat. Commun.* **7,** 13125 doi: 10.1038/ncomms13125 (2016).

## Supplementary Material

Supplementary Information Supplementary Figures 1-13
