# Supplementary material for: Specification of haematopoietic stem cell fate via modulation of mitochondrial activity
Source: Nat Commun. 2016 Oct 12;7:13125. doi: 10.1038/ncomms13125 (PMC5064016; doi:10.1038/ncomms13125)
Supplement: Supplementary Information — Supplementary Figures 1-13 [file ncomms13125-s1.pdf]

Supplementary Figures

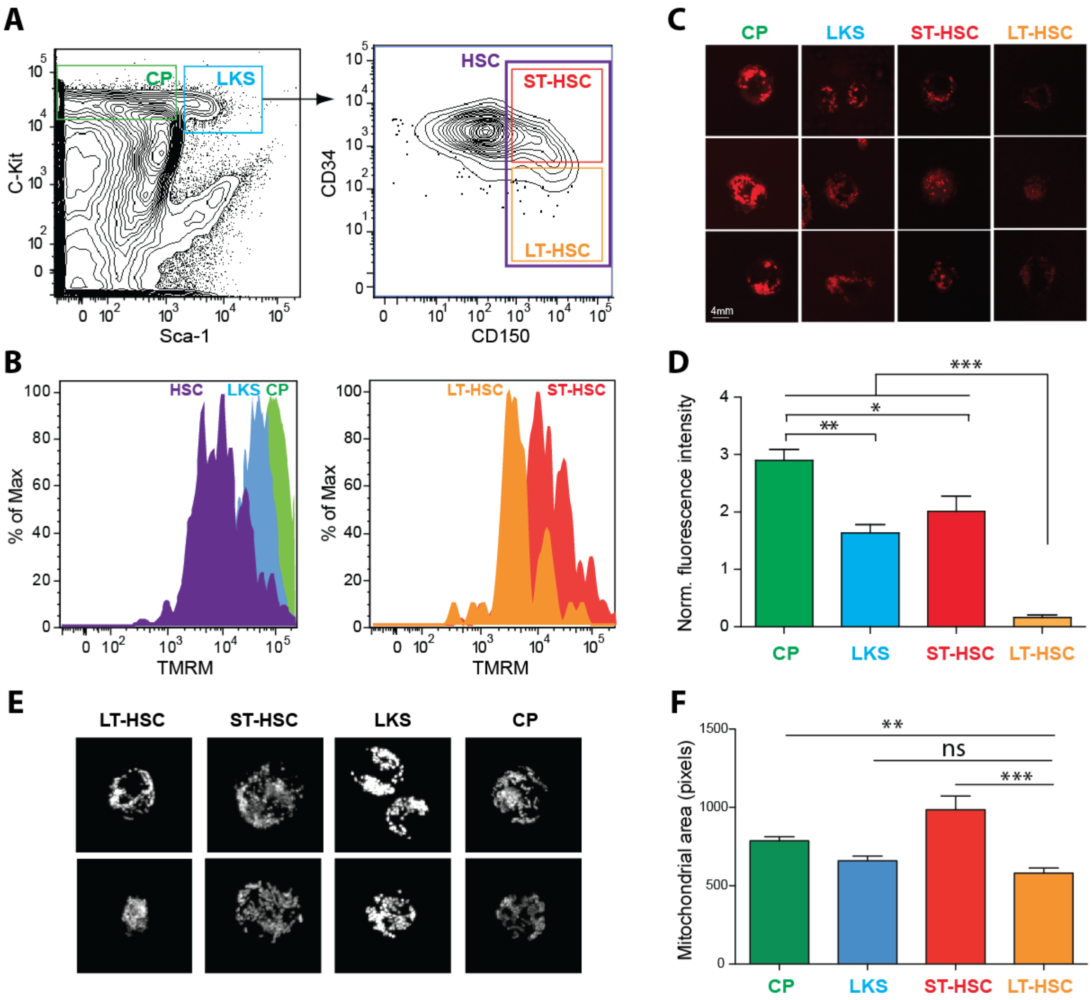

Supplementary Figure 1: Phenotypically defined hematopoietic stem and progenitor cell populations show distinct mitochondrial activity and mass. (**A**) Isolation by FACS of commonly used, phenotypically defined hematopoietic stem and progenitor populations. Committed progenitors, CPs: c-Kit+; LKS: Lin- c-Kit+ Sca-1+ (*i.e.* a population that comprises all multipotent stem and progenitor cells in the bone marrow); short-term HSCs: LKS CD150+ CD34+ (ST-HSC); long-term HSCs: LKS CD150+ CD34- (LT-HSC). (**B**) Flow cytometry analysis of CPs, LKS, ST-HSC and LT-HSC based on ΔΨm labeled with TMRM. Each population is marked by a differential ΔΨm level with a stepwise increase from the most primitive to the most committed population. (**C,D**) Confocal imaging and image analysis of live TMRM-labeled stem/progenitor cells confirms an increase of ΔΨm with increasing commitment level (n=7). Three representative examples are shown for each cell population (**D**). (**E**) Mitochondrial mass (mito-EGFP reporter) in different hematopoietic stem/progenitor cell populations. Images represent maximum intensity projections of the corresponding Z-stacks (0.28 um step). (**F**) Quantification of the mito-EGFP intensity-thresholded area (LT-HSC n=36, ST-HSC n=41, LKS n=77, CP n=113). ***P< 0.001, **P< 0.01 and *P< 0.05

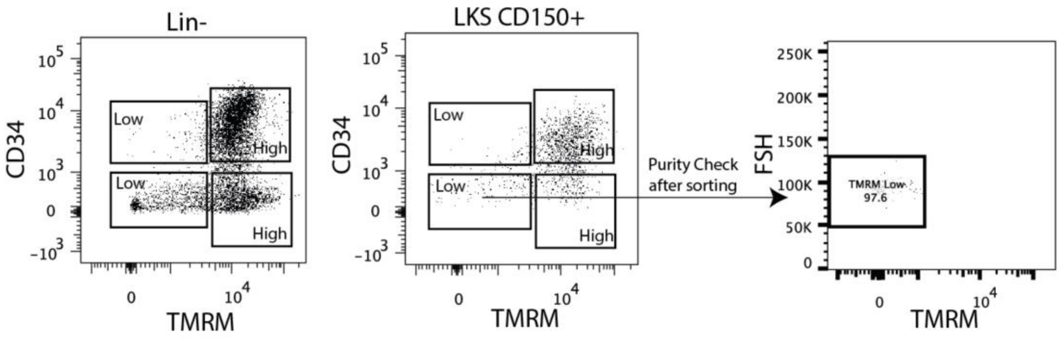

Supplementary Figure 2: Selection of the TMRM^{low} and TMRM^{high} gates. Gates are selected by plotting CD34 vs TMRM in Lin- population. This strategy allowed to define 4 distinct gates CD34+TMRM^{low} and TMRM^{high}, CD34- TMRM^{low} and TMRM^{high} that subsequently are applied to the rare LT and ST populations (LKS CD150+).

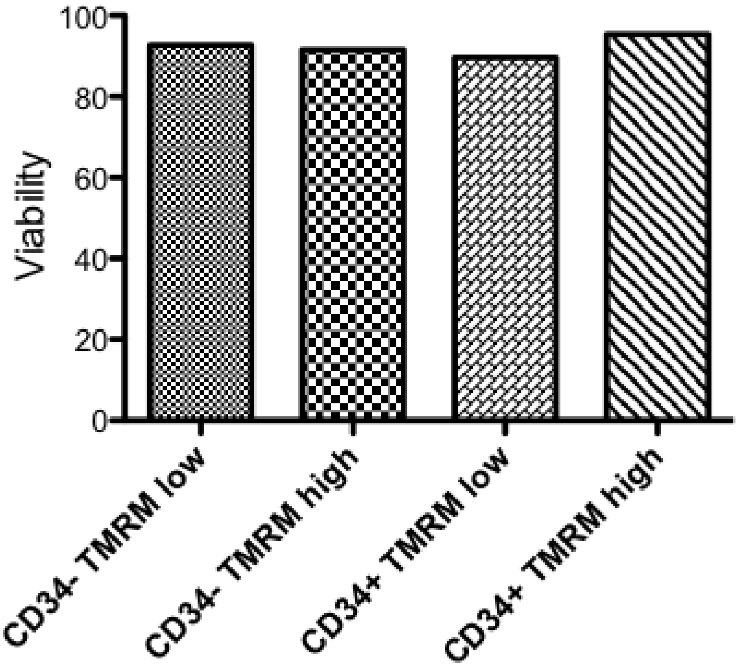

Supplementary Figure 3: Viability analysis of stem/progenitor populations sorted by TMRM intensity. The four indicated populations were sorted and cultured in vitro for 24 hours, followed by Propidium Iodide (PI) staining to assess cell viability. Flow cytometry analysis shows no difference in the PI negative (viable) fraction in the four fractions, ruling out that TMRM staining is related to differences in cell death.

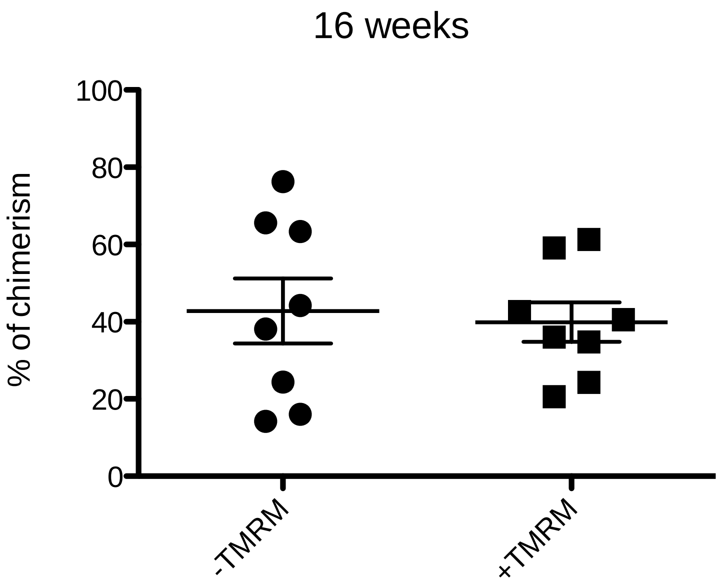

Supplementary Figure 4: Engraftment capability of BM labeled or not labeled with TMRM. Peripheral blood analysis 16 weeks after transplantation doesn't show differences in engraftment capability of BM stained +/- TMRM.

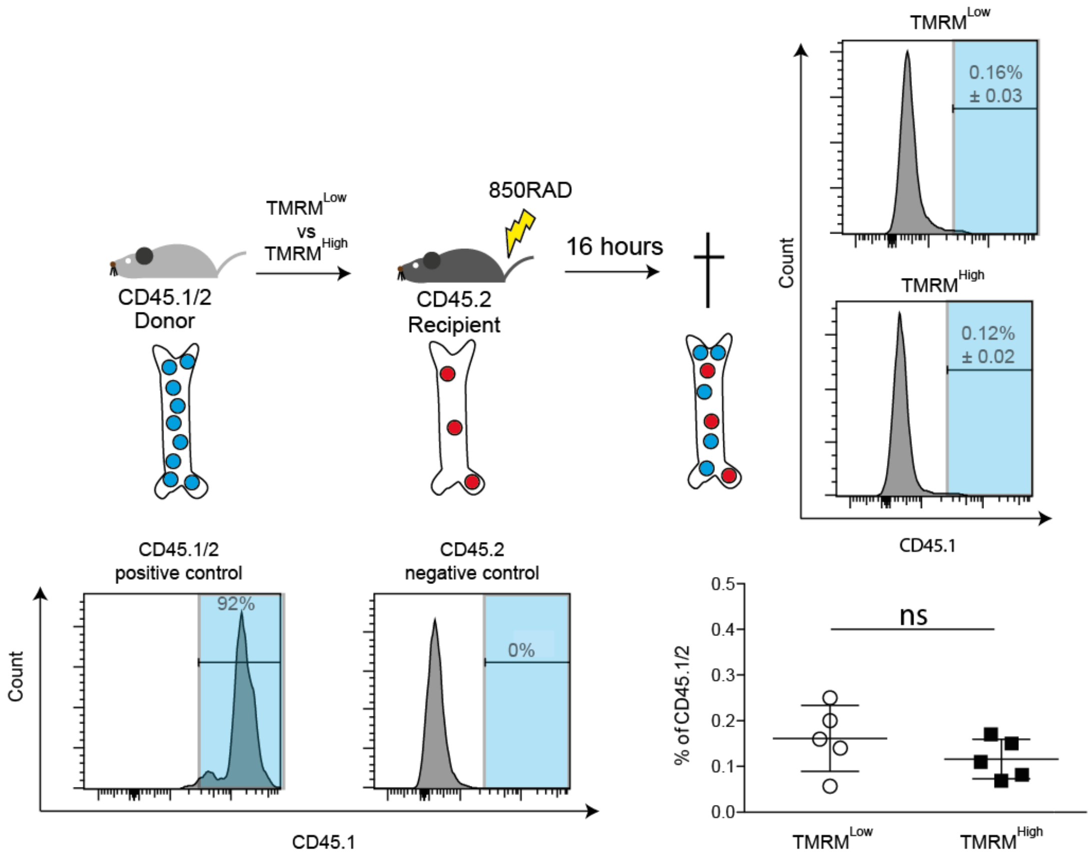

Supplementary Figure 5: Comparison of homing capacity of LT-HSC:TMRM^{low} vs. **LT-HSC:TMRM^{high}.** 4000 LT-HSC:TMRM^{low} and TMRM^{high} were injected in lethally irradiated mice and their presence in the BM was assessed 16 hours post-transplantation by flow cytometry analysis.

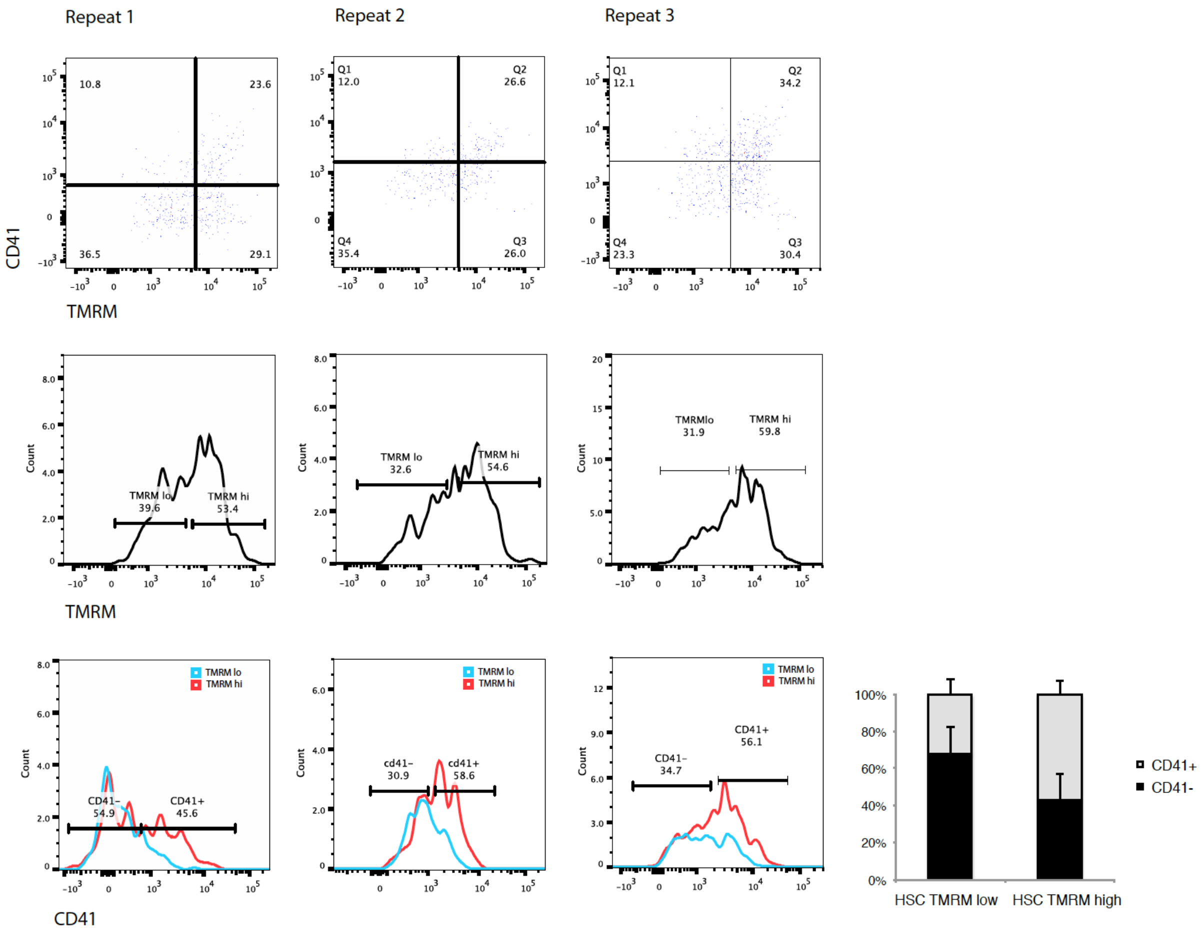

Supplementary Figure 6: CD41- HSCs are enriched in the most primitive LT-HSCs. Flow cytometry plots of three independent experiments showing levels of TMRM in HSC CD41- cells (full line) and HSC CD41+ cells (dashed line).

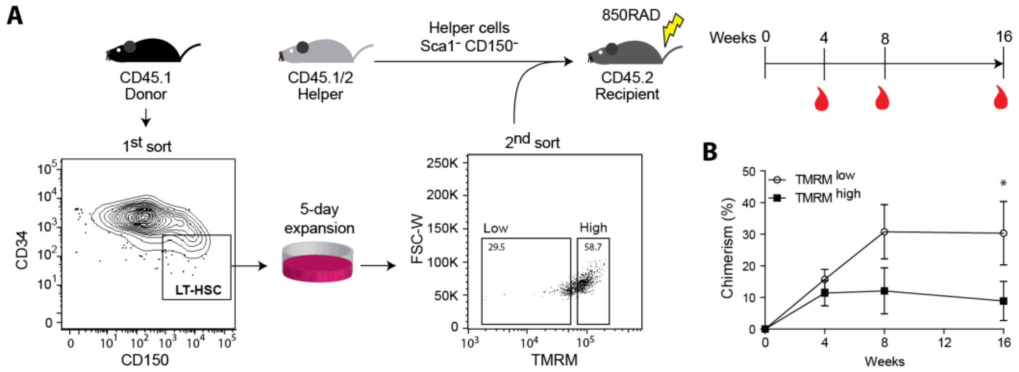

Supplementary Figure 7: A low mitochondrial activity marks self-renewing HSCs in culture. (A) TMRMlow and TMRMhigh cells of 5-day in vitro-expanded LT-HSCs were FACS-sorted and transplanted into lethally irradiated mice together with helper cells (Sca1-, CD150-). Multi-lineage blood reconstitution was measured at 4,8 and16 weeks (n=12 for each condition). (B) The TMRMlow cell fraction of culture-expanded HSC progeny show higher chimerism compared to TMRMhigh cells.

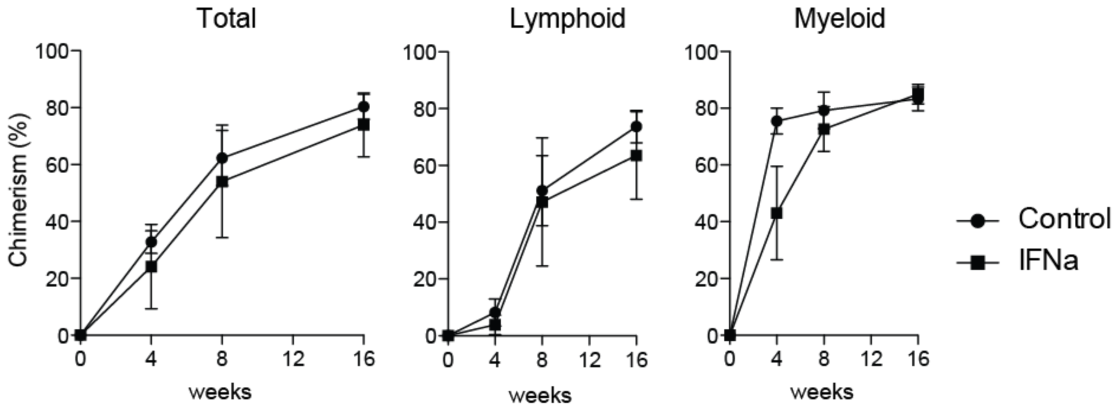

Supplementary Figure 8: IFNalpha activation does not result in loss of stem cell potential. HSCs isolated from control and IFNa-treated mice show similar long-term blood reconstitution levels in lethally irradiated recipients.

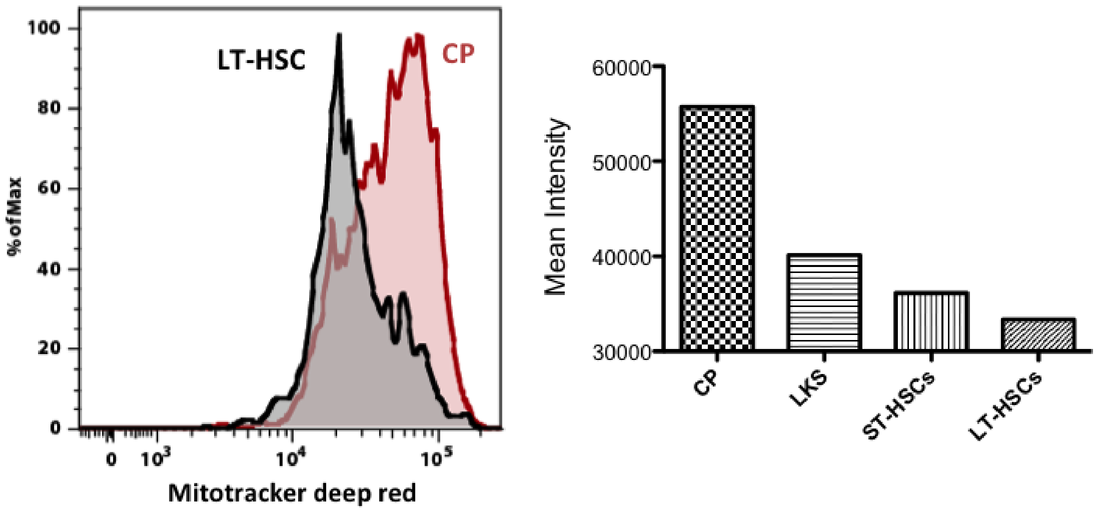

CP: Lin- Ckit+

LT-HSC: LKSCD150+CD48-CD34-

ST-HSC: LKSCD150+CD48-CD34+

Supplementary Figure 9: MitoTracker® Deep Red analysis of different hematopoietic stem and progenitor compartments. Whole bone marrow cells were stained with MitoTracker® (labeling active mitochondria) and HSC markers, and then analyzed by flow cytometry. MitoTracker® staining shows a similar step-wise increase in intensity from the most primitive to most committed population as already demonstrated for TMRM.

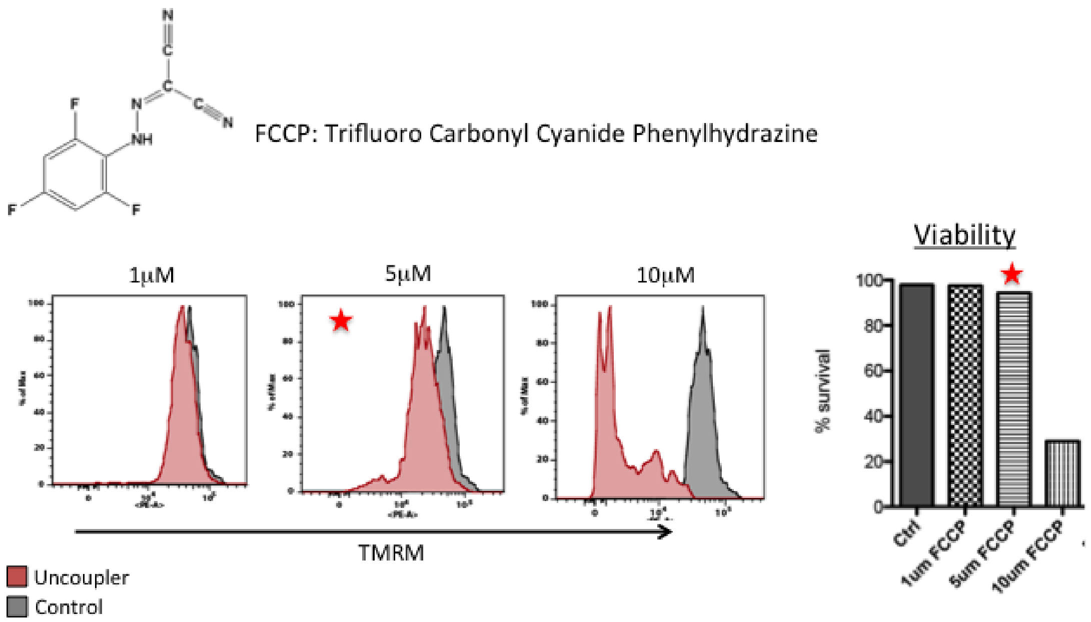

Supplementary Figure 10: Titration of FCCP-mediated uncoupling. Sorted HSCs were cultured in differentiation condition with or without (Ctrl) FCCP for 5 days. FCCP was replenished in the culture media every 24 hours. A concentration-dependent increase in inhibition of mitochondrial activity was seen upon addition of FCCP. A concentration of 5□M lowered mitochondrial activity without affecting cell viability.

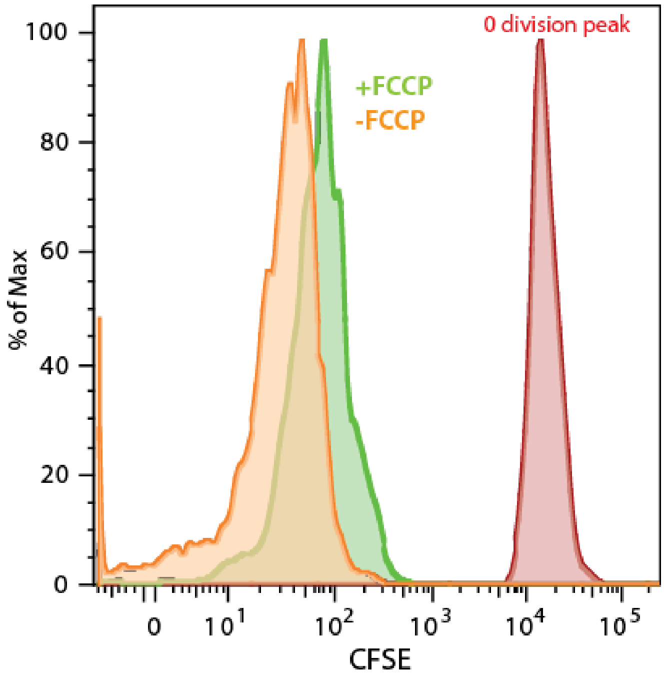

Supplementary Figure 11: CFSE analysis of cultured HSCs in the presence of the uncoupler FCCP. HSCs were isolated from the bone marrow and stained with CFSE followed by a 5-day culture in differentiation condition with or without FCCP. CFSE analysis at the end of culture period demonstrates that all cells had undergone several rounds of division in both conditions, ruling out the presence of quiescent or very slowly dividing cells in both conditions.

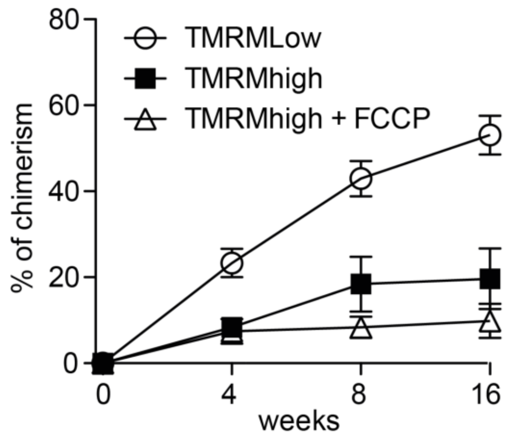

Supplementary Figure 12: TMRM^{high} population treated with FCCP. LT-HSC:TMRM^{high} cells cultured in presence of FCCP don't have enhancement in engraftment has compared to the untreated one, indicating that LT-HSC:TMRM^{high} cells cannot be converted in LT-HSC:TMRM^{low} cells.

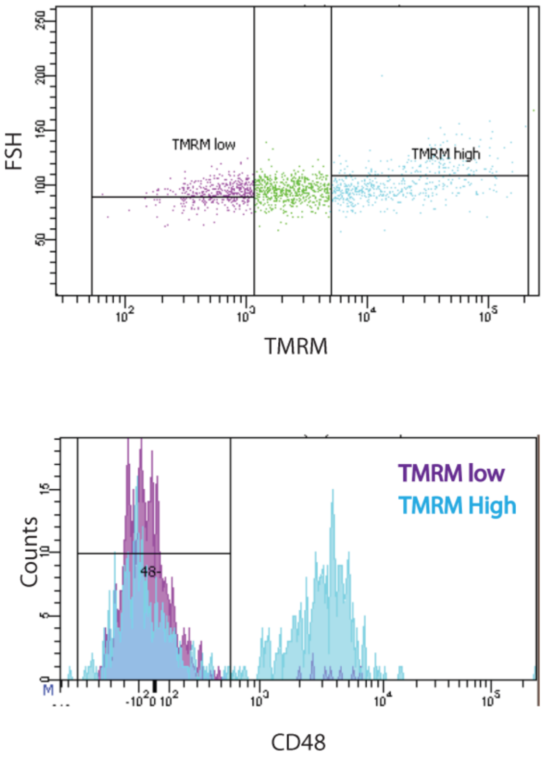

Supplementary Figure 13: Distribution of CD48 SLAM marker on LKS CD150+CD34- TMRM^{Low} vs TMRM^{high}. TMRM low fraction is almost exclusively CD48- while the TMRM high fraction contains both population CD48- and CD48+.
